# Top 10 lessons learned: a retrospective of ASHE careers

**DOI:** 10.1017/ash.2026.10328

**Published:** 2026-04-13

**Authors:** Lara A.C. Phipps, Sarah A. Baig, Priya Nori, Gonzalo Bearman

**Affiliations:** 1 Cornell University, USA; 2 Virginia Commonwealth University School of Medicine, USA; 3 Albert Einstein College of Medicine, USA

## Abstract

As Antimicrobial Stewardship and Healthcare Epidemiology (ASHE) concludes its first five years of existence, we reviewed and summarized the career perspectives publications. ASHE Careers publications are structured interviews with luminaries in infection prevention, public health, and antimicrobial stewardship, where leadership and professional development themes are individualized and explored. Across 14 published interviews from experienced infectious disease professionals identified, we synthesized 10 important themes amongst them. Among them emerged a strong emphasis on the central role of mentorship in career development, the importance of maintaining adaptability, and willingness to explore emerging opportunities of an ever-changing field. Together, these lessons offer guidance for those seeking direction or growth in healthcare epidemiology.

## Introduction

Through examining the career paths and personal anecdotes of numerous leaders in infectious disease and healthcare epidemiology, common themes and lessons emerge that help paint the story of how these successful leaders came to be. Many of them credit their accomplishments to the mentors who offered guidance and encouragement during challenging times. Finding fulfillment through mentorship with others, passions outside of work, and maintaining a growth mindset have helped keep these successful physicians grounded through what can be an unpredictable career path. This willingness to explore not only new career paths but also the opportunities and emerging technologies within them is commonly cited as one of the most important drivers of success within epidemiology. Neither the impact of their actions on the people around them nor the physical environment surrounding them can be ignored. Each of these factors plays a vital role in shaping the field of epidemiology today and serves as a lasting lesson for those entering and growing within it. We summarize the top ten lessons of the last 5 years from the Careers section of *Antimicrobial Stewardship and Healthcare Epidemiology* (ASHE) (Table 1).

**Table 1.** Top 10 lessons learned from ASHE career

Lesson	Key points
1. Importance of supportive mentors and trusting in their guidance	Mentors can recognize potential and qualities that may not be apparent to you, and thus guide you to professional achievements Mentors play a supportive role when doubt creeps in, and progress feels difficult
2. The fulfillment in becoming a mentor to others	Mentors find fulfillment in celebrating the success of their mentees and the personal relationships created Mentees bring fresh ideas and perspectives that help advance their mentor’s research and career
3. The importance of responsible resource management	Excessive use of disposable items in healthcare has significant environmental impacts Lack of awareness regarding labor conditions in factories where healthcare items are sourced
4. Embracing the potential of new technologies	AI is already being implemented in healthcare to assess individual patients’ antibiotic risk profiles AI may significantly benefit patients by evaluating existing data more efficiently
5. Maintaining an open mindset to new professional pathways and opportunities	Value of making bold decisions and being open to new opportunities Stepping away from positions or paths that don’t align with your goals and trusting that direction
6. The importance of an understanding of statistics to healthcare epidemiology	Establish a foundational understanding of statistics Develop meaningful and sustained statistical collaborations before and during the research project
7. The importance of maintaining a work-life balance	Setting firm boundaries and saying no to preserve longevity Maintaining passions and hobbies for long-term success and fulfillment
8. Adversity fuels growth and resilience	-unexpected challenges can guide individuals to meaningful and fulfilling paths trial and error cultivate resilience and prepares for success
9. Trust is built through openness and honest communication.	clear communication is essential for trust in science and public health trust depends on forging connections and acknowledging uncertainty
10. Lifelong learning drives personal and professional growth	challenging assumptions promotes growth and discovery academic and professional flexibility allows exploration of ideas and sustains curiosity

## The importance of having supportive mentors and trusting in their guidance

Mentors share two very important roles with mentees. One is their ability to see the bigger picture, to recognize qualities and potential in you, even when you can’t see them yourself. When Dr. Pittet set his sights on a career in critical care, a wise mentor told him, “You need to do internal medicine, then, I want you to do infectious disease.”^1^ Despite some hesitation, he followed that advice, which led to a global career and over 500 publications. That same advice led him to the introduction of alcohol-based hand rubs at the bedside, resulting in a 50% reduction in healthcare-associated infection (HAI) rates. He shared these findings worldwide and earned recognition through the World Health Organization’s first Global Patient Safety Initiative for Hand Hygiene Promotion. This success inspired him to establish the International Consortium for Prevention & Infection Control, which has fostered global collaboration and grown into a leading conference in infection prevention.^1^

Secondly, mentors also play a vital supportive role when doubt inevitably creeps in, especially in the early phases of a career. Dr. Marisa Holubar credits her mentor with gently pushing her toward her goals when she felt stuck.^2^ Similarly, Dr. Cawcutt credits her mentors as some of the first to believe in her goals, helping her navigate the challenges of gender bias, burnout, and skepticism on her nontraditional path to becoming an infectious disease physician.^3^

## The fulfillment in becoming a mentor to others

Perhaps less recognized in the role of mentorship is the benefits a mentor gains from their relationship with the mentee. As Dr. Wenzel explains, a mentor is someone who selflessly celebrates the successes of their mentee and advocates for their autonomy and creativity, ultimately serving to protect their mentee’s time.^4^ Becoming a mentor can provide a deeper sense of meaning in one’s work through the close personal relationships that mentors build with mentees and the rewarding experience of seeing them thrive.^5^ Individuals with unique interests and energy shape each mentoring relationship, which can, in turn, help propel the careers of both the mentor and the mentee. As Dr. Herwaldt reflects, “Graduate students, ID fellows, and postdoctoral fellows have enriched my career by enabling me to do studies that I wouldn’t have been able to do on my own.”^5^ Similarly, Dr. Judith Guzman-Cottrill agrees that when mentees bring fresh ideas and energy to the relationship and mentors offer guidance, both experience increased opportunities for career advancement and personal fulfillment.^6^

## The importance of responsible resource management

A very often overlooked aspect of the field of infectious disease is how the resources used to keep patients safe have impacts far beyond the healthcare field. As Dr. Mahmood Bhutta points out, healthcare relies on excessive use of disposable products. For example, the routine use of products like gloves when proper handwashing would suffice, use of products designed for single-use (gowns, endoscopes, etc.) when sterilization techniques could be possible without impacting patient outcomes. Bhutta further notes that beyond the environmental waste created by single-use products, these items are often used without consideration or understanding of their sourcing. Many of these products (gloves, surgical instruments, etc.) are manufactured in factories where labor conditions are exploitative, a reality that has only recently begun to receive scrutiny and be considered when choosing the suppliers of these products. Although patient safety is always the priority, the environmental and sociological effects that products have cannot continue to be ignored when considering how and when to use them.^7^

## Embracing the potential of new technologies

Although the future impact of artificial intelligence (AI) on epidemiology remains uncertain, its potential to accelerate research and enhance patient safety is evident. Dr. Vilar-Compte advises young professionals entering the field to stay curious and attentive to developments in AI, emphasizing its likely transformative role in the future of medicine.^8^ A specific example of how this is already coming to light is the work of Susan Huang, Shruti Gohil, and colleagues, who developed a real-time system within the Hospital Corporation of America’s electronic medical record (EMR) infrastructure to assess individual patients’ antibiotic resistance risk profiles. This AI-driven tool has already significantly reduced the use of broad-spectrum antibiotics. Dr. Srinivasan expresses strong optimism about the project, highlighting the excitement of being able to use existing data more effectively. He envisions a future in which AI continuously extracts and analyzes patient data in real time to inform and improve clinical decision-making.^9^

## Maintaining an open mindset to new professional pathways and opportunities

Many of the most prominent and fulfilled epidemiologists did not intend to enter the field or pursue the specific opportunities that shaped their careers. However, through their willingness to seize opportunities as they arose, follow their passions as they developed, and listen to the guidance of their mentors, many of these epidemiologists achieved both great success and deep personal fulfillment. While acknowledging that change can be daunting and risky for those in medicine who believe they have their lives planned out, some of the strongest advice given by both Dr. Mike Edmond and Dr. Daniel J. Sexton to young ID specialists is to be brave enough to make bold decisions and embrace change despite the uncertainty.^10^ This includes stepping away from positions or paths that don’t align with your goals. For instance, Dr. Suzanne Bradley declined the opportunity to become a healthcare epidemiologist twice in favor of a research-focused career. However, by adapting to changes in her lab, her career eventually led her back to healthcare epidemiology. She would not have had these opportunities had she not remained open to changing direction throughout her career.^12^ Dr. Herwaldt, who served as an Epidemic Intelligence Service officer in the CDC’s Respiratory and Special Pathogens Division and later completed an infectious disease fellowship, found a unique way to improve communication and understanding between doctors and their patients simply by participating in a writing workshop and being open to what it inspired her to do. This workshop, along with her willingness to explore uncharted territory, led her to publish *Patient Listening: A Doctor’s Guide*, a project she never could have imagined pursuing.^5^

## The importance of an understanding of statistics to healthcare epidemiology

Although epidemiologists may not be required to employ complex statistical methodologies, they can overlook the importance of having a general understanding of these techniques. Hospital epidemiologist Dr. Diana Vilar-Compte and Dr. Richard Wenzel both strongly encourage those interested in epidemiology to acquire statistical knowledge in order to be successful in the field.^4,8^ Dr. Wenzel urges epidemiologists to aim for “fluency in understanding statistics, their proper use, and knowledge of the underlying assumptions of statistical formulas and models.”^13^ He stresses the importance of learning statistics, whether in a formal classroom setting or through informal means, to understand and critically evaluate options for statistical tests. He also highlights the need for establishing meaningful and sustained relationships with statisticians. Dr. Wenzel recommends engaging with a statistician both before beginning research and throughout the process to ensure efficient data analysis and the effectiveness of the research.^13^

## The importance of maintaining a work-life balance

Crucial to longevity and success in epidemiology is finding a way to maintain a work-life balance that not only allows for professional success but also fosters fulfillment outside the profession. Early in an epidemiology career, it can be incredibly easy to overwork and neglect personal needs because of the numerous opportunities that arise and the hesitation to let them pass by. However, Dr. Holubar cautions against this, reflecting on her own experience of overextending herself early on. She now advises others to first clarify their values and priorities to critically evaluate which opportunities are right for them. She emphasizes pacing oneself by focusing only on the opportunities most aligned with personal and professional values, while maintaining clear personal boundaries with work.^2^ Similarly, Dr. Lilian Abbo credits her grounded attitude and positivity to the things she loves outside of work, including gardening, workouts, and world traveling, as well as the professional boundaries she maintains.^13^ Clearly, saying no to certain opportunities and making space for life outside of epidemiology can be crucial for achieving long-term career success and personal fulfillment.

## Adversity fuels growth and resilience

Adversity is often the foundation upon which growth and resilience are built. Early in his career, Dr. Ramanan Laxinarayan attempted to organize the cleanup of two severely polluted rivers in Chennai, the city where he grew up. As he reflects, “What I soon found out was that the reason the rivers were polluted with sewage was not because the city didn’t know how to treat its sewage, but rather because the poorest residents of the city who lived on the banks of the river had no voice and no ability to influence the city’s priorities.”^14^ This insight revealed the limits of technical expertise alone and prompted him to seek skills in leadership and advocacy that extended well beyond his engineering training.

Career paths shaped by adversity are rarely linear. Dr. Daniel J. Sexton captures this reality when he notes that “Although there is no rewind button in life, detours are allowed and, as in my case, detours may sometimes be surprisingly beneficial,”^10^ highlighting how unexpected turns can lead to meaningful and unanticipated outcomes. Similarly, Dr. Marisa Holubar notes the importance of trial and error, particularly in the early stages of one’s career, explaining that “you don’t have sufficient context to evaluate what opportunities are right for you until you’ve tried some that aren’t the best fit.”^2^ Experiences that initially feel like missteps often provide the clarity needed to make more informed choices later.

Even intellectual adversity plays a vital role. Dr. Richard Wenzel emphasizes that “successful people accept debate comfortably, welcome differences of opinion, and remain open to challenge.”^4^ In this way, disagreement and discomfort can sharpen thinking, encourage growth, and strengthen collaboration beyond complacency.

Seen through these lived experiences, adversity is not an obstacle to success but a central driver of it. The detours, debates, and disappointments are not roadblocks but catalysts for long-term success in epidemiology and public health.

## Trust is built through openness and honest communication

Trust is built through openness and honest communication. In medicine and public health, trust is not automatic; it must be actively earned through transparency, accountability, and meaningful engagement. This is especially critical when scientific evidence is intended to inform public policy. As Ramanan Laxminarayan emphasizes, “If one is doing work that should inform the view of policymakers, then it is incumbent on academics to get out of their comfort zone and communicate to a broader audience.”^14^ Scientific rigor alone is insufficient if findings remain confined to academic journals and inaccessible to those who shape policy or to the communities most affected by those decisions. Researchers, therefore, have a responsibility not only to generate evidence but also to communicate it clearly, honestly, and in a way that is understandable to non-experts.^14^ Such openness fosters mutual understanding, counters misinformation, and creates a foundation of trust between scientists, policymakers, and the public, an essential prerequisite for evidence-based decision-making.

In clinical practice, trust is equally dependent on relationship building as it is on expertise. Dr. Marisa Holubar emphasizes that “influencing behavior change, building relationships, and connecting with diverse audiences” are core to effective antimicrobial stewardship.”^2^ This underscores that trust is not established through authority alone, but through sustained engagement with clinicians, patients, and interdisciplinary healthcare teams across varied settings. At the same time, trust requires humility and openness. As Arjun Srinivasan explains, “I think part of good leadership is modeling that vulnerability for people, so they feel more comfortable voicing their frustrations.”^9^ Acknowledging uncertainty in this way does not weaken a clinician’s position; rather, it strengthens credibility, encourages honest dialogue, and fosters mutual respect—foundations that are essential for sustained trust and effective clinical decision-making.

Finally, trust in mentorship is strengthened when leaders are willing to share not only their successes but also the uncertainties inherent in professional growth. As Dr. Wenzel reflects, “Learn to be comfortable with uncertainty and change. Plan for it. Hope for it. Embrace it.”^4^ This openness humanizes leadership, fosters more authentic mentor–mentee relationships, and reinforces that growth often emerges through adaptation rather than linear progress.

Together, these perspectives illustrate that trust cannot be manufactured; it is cultivated through honest communication, humility, and genuine relationship-building. Whether engaging with policymakers, collaborating with colleagues, or mentoring the next generation, trust becomes the cornerstone of meaningful and lasting impact in epidemiology.

## Lifelong learning drives personal and professional growth

Lifelong learning is less about accumulating credentials and more about cultivating the mindset that growth never really stops. Dr. Ramanan Laxminarayan recalls advice from his advisor that reshaped how he viewed an academic career: “The great advantage of academia was that one could think about whatever one chooses to think about, and that flexibility is a tremendous blessing.”^14^ That freedom, he realized, was not just a professional privilege but a way to keep curiosity alive and thinking expansive.

Yet curiosity does not sustain itself—it must be intentionally cultivated. Dr. Richard Wenzel frames expertise in epidemiology not as a fixed destination but as a continuous practice of inquiry, noting that “mastering the field requires you to remain curious, to continually look at the challenges in epidemiology, to discuss and debate the approaches to solutions.”^4^ He further emphasizes that progress depends on surrounding oneself with people “who love ideas, who can challenge their own assumptions and current dogma, then ask why they should accept what is considered common sense.”^4^ In this sense, comfort with disagreement becomes a driver of discovery rather than a source of friction.

The importance of continual learning becomes especially evident during moments of crisis. Dr. Judith Guzman-Cottrill reflects on her experience during the 2020 Oregon wildfires, when emergency evacuations had to be coordinated alongside ongoing COVID-19 restrictions. “I was involved in emergency meetings to quickly modify COVID-19 mitigation requirements to allow for fire-related evacuations,” she recalls, adding simply, “I am always learning.”^6^ Navigating rapidly evolving circumstances under intense pressure reinforced that professional growth is not limited to structured training but is often shaped most profoundly by the need to adapt in real time to unforeseen challenges.

Viewed through these varied lenses—academic, intellectual, and practical—lifelong learning emerges as a constant force rather than a finite phase. It is the thread that ties together academic freedom, intellectual debate, and real-world experience. To keep learning is to remain open, adaptable, and resilient—qualities that not only advance professional excellence but also sustain long-term personal fulfillment.

## Conclusion

The most impactful careers in infectious diseases and healthcare epidemiology are rarely linear, but they are consistently intentional. The leaders profiled in this retrospective did not follow a singular blueprint for success; instead, they cultivated adaptability, embraced uncertainty, and remained anchored by purpose. Whether navigating mentorship, responding to adversity, or engaging with emerging technologies such as artificial intelligence, each person’s journey reflects a deep commitment to both scientific rigor and human connection.

These lessons do not prescribe a rigid path forward but offer a map of diverse values—curiosity, humility, resilience, and integrity—that guide meaningful work. The next generation of epidemiologists will face new challenges, but the wisdom of those who came before remains relevant: growth is iterative, influence is relational, and the greatest contributions often arise not from perfect plans but from bold pivots, hard-won insights, and the courage to lead differently. In a field defined by uncertainty, these enduring lessons offer both grounding and inspiration for what comes next.
